# Implant Surfaces Containing Bioglasses and Ciprofloxacin as Platforms for Bone Repair and Improved Resistance to Microbial Colonization

**DOI:** 10.3390/pharmaceutics14061175

**Published:** 2022-05-30

**Authors:** Irina Negut, Carmen Ristoscu, Tatiana Tozar, Mihaela Dinu, Anca Constantina Parau, Valentina Grumezescu, Claudiu Hapenciuc, Marcela Popa, Miruna Silvia Stan, Luminita Marutescu, Ion N. Mihailescu, Mariana Carmen Chifiriuc

**Affiliations:** 1 National Institute for Lasers, Plasma and Radiation Physics, 409 Atomistilor Street, 077125 Magurele, Romania; negut.irina@inflpr.ro (I.N.); tatiana.alexandru@inflpr.ro (T.T.); valentina.grumezescu@inflpr.ro (V.G.); hapenciuc.claudiu@inflpr.ro (C.H.); ion.mihailescu@inflpr.ro (I.N.M.); 2 Extreme Light Infrastructure-Nuclear Physics, Horia Hulubei National Institute for R&D in Physics and Nuclear Engineering, 077125 Magurele, Romania; 3 National Institute of Research and Development for Optoelectronics-INOE2000, 409 Atomistilor St., 077125 Magurele, Romania; mihaela.dinu@inoe.ro (M.D.); anca.parau@inoe.ro (A.C.P.); 4 Microbiology Immunology Department, Faculty of Biology, University of Bucharest, 077206 Bucharest, Romania; marcela.popa@bio.unibuc.ro (M.P.); luminita.marutescu@bio.unibuc.ro (L.M.); 5 Department of Biochemistry and Molecular Biology, Faculty of Biology, University of Bucharest, Splaiul Independentei 91-95, 050095 Bucharest, Romania; miruna.stan@bio.unibuc.ro (M.S.S.); carmen.chifiriuc@bio.unibuc.ro (M.C.C.); 6 Department of Microbiology, Faculty of Biology, University of Bucharest, Aleea Portocalelor Str. 1-3, District 5, 060101 Bucharest, Romania; 7 Romanian Academy of Scientists, 3 Ilfov Str., District 5, 050044 Bucharest, Romania; 8 The Romanian Academy, Calea Victoriei 25, District 1, 010071 Bucharest, Romania

**Keywords:** thin film, antibiotic delivery, biocompatibility, bioglass, laser deposition

## Abstract

Coatings are an attractive and challenging selection for improving the bioperformance of metallic devices. Composite materials based on bioglass/antibiotic/polymer are herein proposed as multifunctional thin films for hard tissue implants. We deposited a thin layer of the polymeric material by matrix-assisted pulsed laser evaporation—MAPLE onto Ti substrates. A second layer consisting of bioglass + antibiotic was applied by MAPLE onto the initial thin film. The antimicrobial activity of MAPLE-deposited thin films was evaluated on *Staphylococcus aureus*, *Enterococcus faecalis*, *Escherichia coli*, and *Pseudomonas aeruginosa* standard strains. The biocompatibility of obtained thin films was assessed on mouse osteoblast-like cells. The results of our study revealed that the laser-deposited coatings are biocompatible and resistant to microbial colonization and biofilm formation. Accordingly, they can be considered viable candidates for biomedical devices and contact surfaces that would otherwise be amenable to contact transmission.

## 1. Introduction

Accidents, falls or direct strikes to the body, deficiency of particular nutrients, persistent bone and skeletal illnesses, or injuries while conducting daily tasks are all possible causes of bone fractures. As bones protect humans’ vital organs like the heart, lungs, and brain, fractures must be treated as quickly as possible [1]. Metallic implants such as bone plates, rods, nails, and other compression plates offer artificial support when the fracture is severe. These devices provide mechanical stability, allowing for optimal bone alignment and function during physiologic loads. As a result, implants alleviate pain, restore mobility, and easily resume the human body’s functions [2].

Implants and bone healing are intimately linked, therefore the stabilized bone should “self-heal” [2]. Major advances have been made in recent years in the understanding and design of implantable devices, notably in the areas of biocompatibility, bioavailability, and efficacy [3]. Several synthetic biomaterials have been designed as substitutes for natural bones. Among them, bioactive glasses (BG) are the most used owing to their biocompatibility and their capacity to form a bone-like mineral phase when in contact with the native hard tissue [4].

Any device implanted into the human body is prone to infections caused by bacteria (e.g., *Staphylococcus aureus*, *Enterococcus faecalis*, *Escherichia coli*, *Pseudomonas aeruginosa*), which further affect bone healing or ingrowth, nonunion of fractured bones, and even implant loosening [5]. These infections are difficult to treat, and they frequently necessitate further surgical procedures such as implant replacement. In the worst-case scenario, they can also result in amputation or death, posing a significant societal and economic burden [6].

For an implant-associated infection to occur, bacteria must first adhere to the implant surface. These infections anchored to the surface are known as biofilms. Biofilms are formed by complex groups of microbial cells of both Gram-positive and Gram-negative bacteria, generating a protective extracellular polysaccharide matrix [7]. Removal of these communities is challenging since biofilms are resilient to the host’s immune system and antibiotic treatment [8].

The dose of systemically administered antibiotics does not often reach the site of infection; bones are moderately perfused organs and the blood flow in infected bone tissues is usually reduced [5]. Locally administered antibiotics for the treatment of bone infections have several advantages over systemic administration, such as the reduction of adverse effects and the risk of overdose while boosting the drug’s bioavailability effectively reaching the target site. Therefore, an implant surface must meet the requirement of a multifunctional system that responds to microbiological signals by releasing antimicrobials or other alleviating compounds [1]. As a result, antimicrobial surfaces are considered as “contact killing” and antimicrobial agent eluting surfaces. Many studies have been reported on antimicrobial coatings developed for the treatment of implantable medical device-associated infections [9,10,11].

Matrix-assisted pulsed laser evaporation (MAPLE) has been applied for the growth of high-quality thin films for an assortment of medical devices, with different biological and antimicrobial functionalities [12,13,14].

Our previous BG-related work demonstrated that MAPLE is a suitable technique for fabricating biocompatible and bone-healing thin films containing combinations of BG and antimicrobial compounds, including doxycycline [15], Neem [16], turmeric and holy basil [17]. These films showed a high antibacterial efficiency against Gram-positive and Gram-negative bacterial strains, and this activity was mediated by a contact-killing effect.

We report herewith composite coatings based on a polymer (Poly(methyl methacrylate)), BG and carboxyfluoroquinoline antibiotic (ciprofloxacin) obtained by MAPLE in order to: (i) reduce corrosion of implant materials; (ii) increase the biocompatibility of implants by promoting bone regeneration and growth, without toxic effects, and (iii) inhibit microbial biofilm formation.

## 2. Materials and Methods

### 2.1. Materials

Chemicals, namely chloroform (CHCl_3_), analytical grade acetone (C_6_H_6_O), ethanol (C_2_H_5_OH), and reagents required for the preparation of the simulated body fluid (SBF), such as NaCl, NaHCO_3_, KCl, K_2_HPO_4_·3H_2_O, MgCl_2_·6H_2_O, HCl, CaCl_2_, Na_2_SO_4_, and (CH_2_OH)_3_CNH_2_ were acquired from Sigma-Aldrich Chemie GmbH (Steinheim, Germany). SBF with an ionic composition identical to blood plasma was prepared according to the corrected Kokubo’s SBF similar to that of human blood plasma [18] by mixing the reagents, following the precise order and quantity.

Grade 4 titanium (Ti) of (10 × 10) and (5 × 5) mm^2^ disks were used as deposition substrates. Preceding the deposition, the disks were cleaned with acetone, ethanol, and deionized water in an Elma X-Tra 30 H ultrasonic bath (Elma Schmidbauer GmbH, Singen, Germany). The choice of Ti is determined by the well-known compatibility of this metal with bone which justifies its intensive use as a biomaterial for designing implantable devices [19].

Since the report on its bioactivity, the 45S5 Bioglass^®^ (45S5) has been the most extensively used in biomedical applications, ranging from scaffolds for tissue engineering, bone grafts, periodontal applications, to coatings for bioinert implants ([20] p. 5). Its composition can be considered as the predecessor of many BG, obtained by removing or adding ions, with the aim of producing materials with novel properties, suitable for specific clinical requirements [21]. Our chosen BG contains 56.5% SiO_2_, 15% CaO, 11% Na_2_O, 8.5% MgO, 6% P_2_O_5_, and 3% K_2_O (in wt%). It is fabricated following the protocol described in references [22,23].

Poly(methyl methacrylate)—PMMA, (C_5_H_8_O_2_)n, is a synthetic biopolymer demonstrating good mechanical stability and chemical (acid and base) resistivity [24]. Due to its inexpensiveness, lightweight features, superior toughness, and cost-effective fabrication, it is widely used in medical applications such as bone cement preparation for drug delivery and release [25], optical systems such as contact lenses [26], or the flat panel industry [27].

Ciprofloxacin (CIPRO), a small molecular drug, is a broad-spectrum antimicrobial carboxyfluoroquinoline agent with demonstrated activity against most strains of Gram-negative bacteria and against certain Gram-positive bacteria [28]. CIPRO proved a powerful antibiotic to be applied for bone infections [29]. CIPRO hydrochloride powder of 99.5% purity was purchased from Gen Hunter (Nashville, TN, USA).

### 2.2. MAPLE Target Preparation and Experimental Conditions

**Figure 1:**
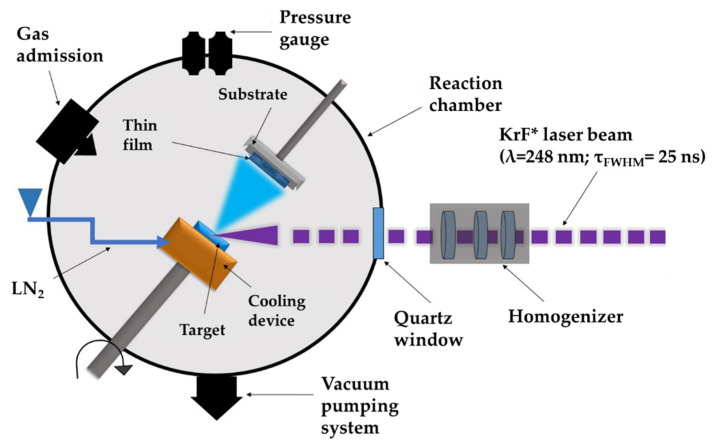
Schematic representation of MAPLE experimental set-up.

MAPLE represents an adjustable laser-based processing method that implies a vacuum deposition chamber where a pulsed laser beam hits and evaporates a rotating cryogenic target. The target is made from a diluted mixture of the complex organic material of interest; the material to be deposited is dissolved in a high vapor-pressure light-absorbent solvent. The volatile solvent is pumped away by the vacuum system. The laser beam transfers softly and without heat and/or biological deterioration and harms diverse compounds, including big molecular weight species, such as polymeric or organic molecules. MAPLE experimental set-up can be seen in Figure 1.

In the first experiment we deposited a thin layer of PMMA by MAPLE onto Ti substrates. A second layer consisting of BG and CIPRO was applied also by MAPLE onto the PMMA initial thin film. The resulting samples are further denoted as BG+CIPRO/PMMA.

For the first MAPLE thin film, 0.6 g PMMA was dissolved into 19.3 mL chloroform to obtain a frozen target in liquid nitrogen. For the second layer, 0.05 g BG and 0.125 g ciprofloxacin were dissolved into 12 mL deionized water and frozen.

The experiments were carried out with a KrF* (λ = 248 nm, τ_FWHM_ ≤ 25 ns) excimer laser source operated at 7 Hz repetition rate and a fluence of 0.55 J/cm^2^. For the deposition of each thin film, 10,000 subsequent laser pulses were applied. In order to obtain a uniform layer and to avoid target drilling, both the substrate and target were simultaneously rotated with 50 rpm, while the background pressure inside the deposition chamber was maintained at 2 × 10^−2^ mbar. Throughout the deposition experiments, the targets were kept at liquid nitrogen temperature using a cryogenic setting. The target to substrate separation distance was set at 5 cm.

### 2.3. Thin Films Characterization

#### 2.3.1. Morphological and Structural Characterization

The surface morphology of samples was inspected by scanning electron microscopy (SEM) using a FEI Inspect S electron microscope (Hillsboro, OR, USA). The investigations were carried out in top-view and cross-section modes, at 20 kV acceleration voltages, in high vacuum, using secondary electron mode. For the reduction of electrical charging through analysis, all samples were capped with a thin gold film.

The surface topography of samples was obtained by atomic force microscopy (AFM), by using an AFM TT-Workshop apparatus (Signal Hill, CA, USA), with a scan area of (15 × 15) µm^2^ and (10 × 10) µm^2^, in non-contact operating mode. Because of the samples’ high roughness, the scanning speed was reduced to 0.5 Hz at 650 mV.

The wettability of the deposited coating was analyzed by the sessile drop method, using an Attension Theta Lite (TL) 101 optical tensiometer (version 1.0.3, Biolin Scientific, Vastra Frolunda, Sweden), in atmospheric conditions (22 °C, 30% relative humidity). The contact angle between the test liquid and surface of the specimens was measured.

The stoichiometry and chemical functions integrity of thin films were studied by means of Fourier-transform infrared spectroscopy (FTIR). The FTIR study was performed using a Nicolet ™ FT-IR iS ™ 50 spectrometer (Thermo Fisher Scientific, Waltham, MA, USA) equipped with an attenuated total reflection (ATR) module, within 3700–700 cm^−1^ range at 4 cm^−1^ resolutions. For the ATR, a ZnSe crystal was used, with the following characteristics: 1.5 mm diameter, one internal reflection at 42° incidence angle, penetration depth of 2.03 µm at 1000 cm^−1^, and 2.4 refractive index. The spectra were taken in absorbance mode, between 1800–650 cm^−1^ with a resolution of 4 cm^−1^. The recorded spectra were averages of 16 scans. FTIR ATR spectra of single components were performed for CIPRO, BG, and PMMA in powder form. The IR spectra of BG+CIPRO/PMMA structures after different immersion times in SBF were recoded.

#### 2.3.2. Drug Release Behavior Evaluation

To simulate the insertion of implants into the human body and the phenomena occurring at the tissue-implant interface as a result of interaction with physiological fluids, BG+CIPRO/PMMA samples were immersed in 1 mL of SBF in glass flasks, at 37 °C, in a Binder microbiological incubator. They were investigated by FTIR.

The volume of the SBF used for drug release evaluation tests was calculated from Equation (1):

Vs = Sa/10(1)

where Vs is the volume of SBF (mL) and Sa is the apparent surface area of the sample (mm^2^).

The SBF solutions containing the released products from thin films were analyzed by UV-Vis after different immersion times. The release profiles of CIPRO over time were recorded by means of an Evolution 220 spectrophotometer (ThermoFisher Scientific, Darmstadt, Germany) within the range 190–1200 nm, in absorbance mode. All measurements were carried out in triplicate, in accordance with ISO/FDIS 23317: 2007 (E). The absorption peaks amplitude, which is proportional to the drug concentration delivered by coatings to the adjacent tissue, was determined.

#### 2.3.3. Electrochemical Investigation

The electrochemical behavior of the investigated samples was analyzed by potentiodynamic polarization and electrochemical impedance spectroscopy (EIS), using a VersaSTAT 3 Potentiostat/Galvanostat system (Princeton Applied Research, Oak Ridge, USA). The measurements were performed in SBF at 37 °C, using a typical three electrode setup with a Pt grid counter electrode (CE) and an Ag/AgCl (saturated KCl) (0.197 V) as the reference electrode (RE). The working electrode consisted of uncoated and BG+CIPRO/PMMA-coated Ti, respectively.

The open circuit potential (EOC) was monitored for 36 h, starting immediately after the sample’s immersion, whereas EIS data was recorded after 1, 12, 24, and 36 h of immersion. The EIS measurements were performed over a range of frequencies (0.2 ÷ 103 Hz), by applying a sinusoidal signal of 10 mV RMS vs. EOC. The data were recorded by VersaStudio software (version 2.60.6, Princeton Applied Research, Oak Ridge, TN, USA) and the EIS fitting procedure was performed using ZView software (version 12136-4, Scribner Associates Inc., Southern Pines, NC, USA).

### 2.4. Biological Evaluation

#### 2.4.1. Biocompatibility Assay

##### Cell Culture

Mouse osteoblast-like MC3T3-E1 cells were grown in Dulbecco Modified Eagle’s Medium (Invitrogen, Carlsbad, CA, USA) with 10% fetal bovine serum (Gibco, Grand Island, NY, USA) at 37 °C in a humidified atmosphere with 5% CO_2_. The cells were seeded at a cell density of 5 × 104 cells/ cm^2^ on the tissue culture plastic surface (TCPS) which served as a control, or on the top of the tested samples (Ti and BG+CIPRO/PMMA) which were previously sterilized under UV light. After 24 h of incubation in standard conditions, the biocompatibility tests were performed.

##### MTT Assay

Cellular viability was measured using the 3-(4,5-dimethylthiazol-2-yl)-2,5-diphenyltetrazolium bromide (MTT; Sigma-Aldrich, St. Louis, MO, USA) assay. At the end of the incubation period, the culture medium was removed and the cells were incubated with 1 mg/mL MTT for 2 h at 37 °C. The purple formazan crystals formed in the viable cells were dissolved with 2-propanol (Sigma-Aldrich, St. Louis, MO, USA) and the absorbance was measured at 595 nm using a plate multireader (GENios Tecan, Ramsey, MN, USA).

##### Griess Assay

Using the Griess reagent, a stoichiometric solution (*v*/*v*) of 0.1% naphthyl ethylenediamine dihydrochloride and 1% sulphanilamide, the level of nitric oxide (NO) released in the culture medium during the 24 h of incubation was measured. Equal volumes of culture supernatants and Griess reagent were mixed and their absorbance was read at 550 nm using the GENios Tecan multireader.

##### F-Actin Staining

At the end of incubation, the cells were fixed with 4% paraformaldehyde for 20 min and permeabilized with 0.1% Triton X-100—2% bovine serum albumin for 45 min. The actin filaments were stained with 10 µg/mL phalloidin-FITC (fluorescein isothiocyanate) and the nuclei were counterstained with 2 µg/mL DAPI (4′,6-diamino-2-phenylindole). The images were obtained with an Olympus IX71 inverted fluorescence microscope.

#### 2.4.2. Antimicrobial Activity Evaluation

The antimicrobial activity of the materials was evaluated on standard strains *Staphylococcus aureus* ATCC 25923, *Enterococcus faecalis* ATCC 29212, *Escherichia coli* ATCC 25922, and *Pseudomonas aeruginosa* ATCC 27853. Following the UV sterilization, the materials were immersed in a 10^5^ CFU (colony forming unit)/mL bacterial suspension and incubated at 37 °C in a shaker. After different periods of time (T0—after initial contact, 15, 30, and 45 min, and 1, 2, 4, and 24 h), the microbial viability was evaluated through serial dilutions plating on nutrient medium (Plate Count Agar, Oxoid, Sigma-Aldrich Chemie GmbH, Taufkirchen, Germany). Controls were represented by bacterial strains cultivated in the same conditions and in presence of Ti in order to evaluate the material’s influence on microbial viability.

## 3. Results and Discussion

### 3.1. Surface Investigation of As-Deposited Structures

**Figure 2:**
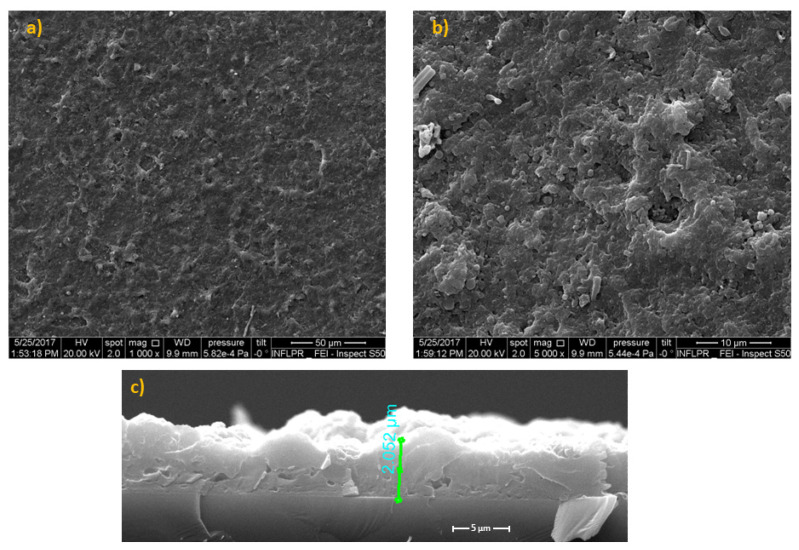
SEM images of BG+CIPRO/PMMA MAPLE films on Si substrates covered with PMMA: (**a**) overview, (**b**) detail, and (**c**) cross-section.

The micro-topography of the thin films is observable from typical SEM images presented in Figure 2. One can note a uniform morphology with spherical, ovoid, or elongated formations of variable dimensions, representative for pulsed laser deposition methods [30]. The micrometric droplets are arbitrarily scattered throughout the surface (Figure 2a). They have a favorable outcome on cell adhesion and growth/proliferation [31]. Considering the magnification and the software of the SEM microscope, estimated thickness for BG+CIPRO/PMMA films was ~2.052 (±5%) µm (Figure 2c).

**Figure 3:**
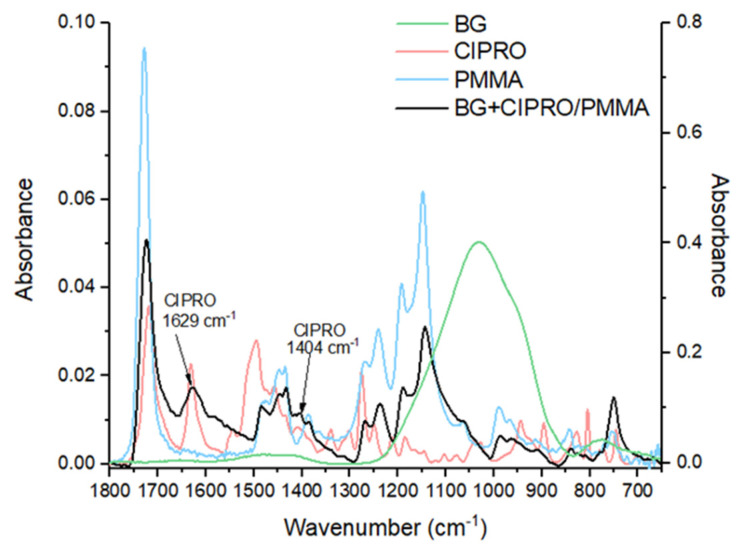
FTIR spectra of BG+CIPRO/PMMA thin film deposited by MAPLE and of single components, namely PMMA, CIPRO, and BG. Note: OY axis (left)—absorbance of CIPRO, PMMA, and BG+CIPRO/PMMA thin film; OY axis (right)—absorbance of BG.

The MAPLE films preserve the composition of raw materials as demonstrated by corresponding FTIR spectra in Figure 3. Peaks belonging to BG, PMMA, and CIPRO in powdery and thin film forms were identified. For the BG+CIPRO/PMMA thin film, FTIR analysis revealed representative peaks for PMMA and CIPRO. PMMA was identified with bands at 1723 cm^−1^ attributed to stretching vibrations acrylate carboxyl groups, at 1447 cm^−1^ and 1431 cm^−1^ attributed to the bending vibration of the C–H bonds of the –CH_3_ groups, and at 1385 cm^−1^ and 751 cm^−1^ responsible for α-methyl group vibrations [32]. The presence of PMMA bands in the IR spectrum suggests that the polymer did not suffer structural modifications during the deposition process. The peak at 1726 cm^−1^ represented by C=O stretching vibration of carboxyl group is characteristic for both CIPRO and PMMA [33,34]. In the case of CIPRO, two typical peaks were identified: at 1629 cm^−1^ attributed to C–O stretching vibration (quinoline) and 1041 cm^−1^ attributed to –CH_2_ deformation vibration [35]. The other CIPRO specific bands between 1500 cm^−1^ and 1000 cm^−1^ overlap the PMMA ones and are not observable. It should be noted that the thin film IR spectrum fingerprint is determined by the weight ratio of PMMA and BG. Thus, for ratios greater than 7.5, PMMA IR peaks predominate, whereas for ratios less than 7.5, higher BG peaks predominate [36]. In our case, the PMMA/BG weight ratio was 12, therefore the IR spectrum of BG+CIPRO/PMMA lacked the characteristic IR bands of BG.

**Figure 4:**
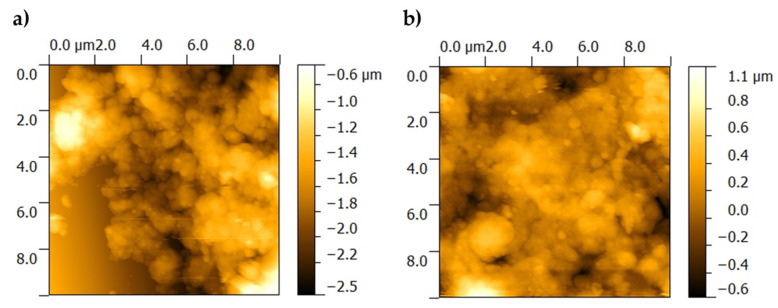
AFM images of as-deposited film surface, at two different locations on the sample. (**a**) BG+CIPRO/PMMA_1 and (**b**) BG+CIPRO/PMMA_2.

The surface topography of the BG+CIPRO/PMMA sample was characterized by a high roughness, which did not allow the scanning of large 15 × 15 µm^2^ areas. In order to avoid contacting the higher peaks present in the topography that could unfavorably affect the image quality, images with an area of 10 × 10 µm^2^ were acquired at two different locations on the sample (Figure 4a,b).

From AFM analyses, one can observe that the BG+CIPRO/PMMA sample (Figure 4a,b) shows high local roughness, reaching peaks of 1 µm. The clusters on BG+CIPRO/PMMA_1 surface contain particles of ~300 nm in size, while the BG+CIPRO/PMMA_2 sample presents large clusters incorporating particles with a diameter of ~200 nm.

AFM data (Table 1) are in good accordance with SEM observations regarding the surface roughness and the presence and dimension of particles.

The surface wettability investigation comprises the contact established between a liquid (in our case SBF) in form of a droplet and a solid (uncoated or MAPLE-coated samples). The contact angle (CA) represents a reliable parameter for biomaterials characterization as tailoring the hydrophobicity/hydrophilicity of surfaces is of great interest for cell adhesion and proliferation.

**Figure 5:**
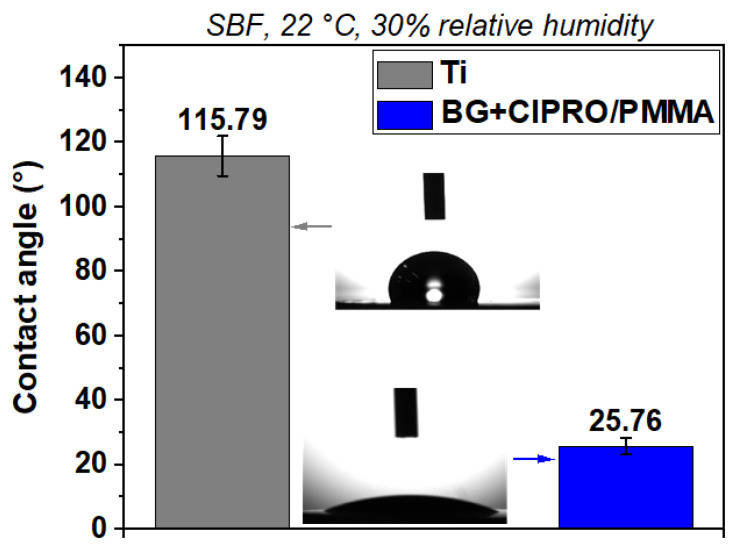
SBF CA measurements on bare Ti and Ti substrate coated with BG+CIPRO/PMMA.

Figure 5 shows representative images of SBF CA as measured on samples. The uncoated Ti substrate was the least hydrophilic and presented a contact angle of 115.79°. The sample coated with BG+CIPRO/PMMA achieved a remarkable improvement in hydrophilicity, with a contact angle of 25.76°. The high wetting of an implant surface increases the bone cells’ contact with an implant, which leads to a rapid and a long-term osseointegration and improves wound healing of hard and soft tissues [37]. As known, hydrophilic properties are influenced by the chemical composition and the surface roughness [38]. As shown in Figure 5, after applying BG+CIPRO/PMMA coating on the substrate, the CA was decreased significantly to 25.76°. One of the possible explanation reasons for the high hydrophilicity of this sample is the presence of the antibiotic. The oxygen functional groups are exposed on the surface of the composite coating, and they act as key sites for water adsorption and ultimately increase the surface wettability. Another factor that influences the wetting properties is the presence of droplets (Figure 2) and particles (Figure 3) on the surface. Roughening the surface has previously been shown to improve the wettability and therefore reduce the contact angle [39,40,41,42].

### 3.2. Characterization of Structures after the Immersion in SBF

**Figure 6:**
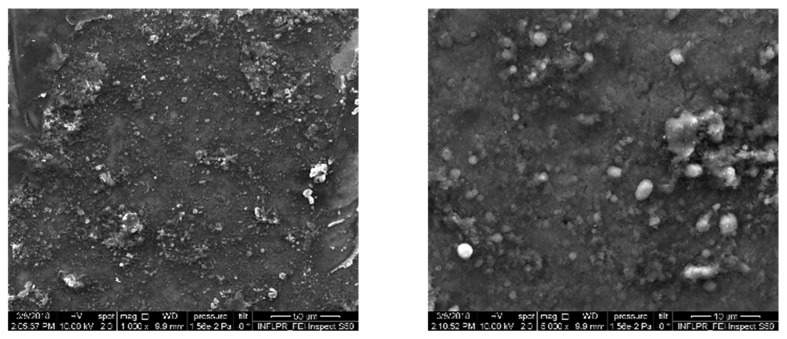
SEM images of BG+CIPRO/PMMA thin films after 21 days of immersion in SBF, at different magnifications.

As observed by SEM images in Figure 6, BG+CIPRO/PMMA thin films deposited on Ti substrates, after 21 days of immersion in SBF, consist of a rather smooth matrix covered with arbitrarily scattered, micronic, isolated, or agglomerated spheroidal particles, probably from the biological apatite.

**Figure 7:**
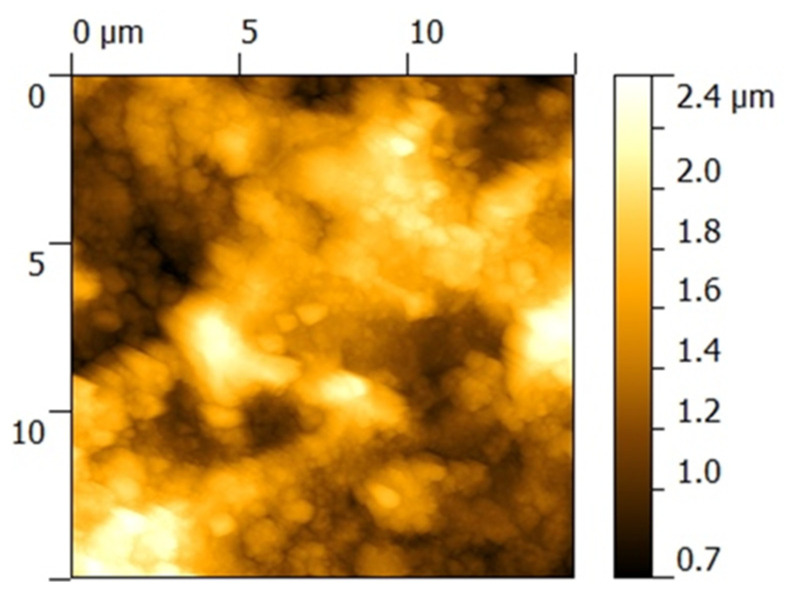
AFM image on 15 × 15 µm^2^ surface areas of BG+CIPRO/PMMA thin films after 21 days of immersion in SBF.

In parallel to SEM, AFM presents 15 × 15 µm^2^ areas with unevenly distributed particles on surfaces (Figure 7). The topology is characterized by an RMS in the range 0.2–0.3 µm (Table 2). The registered values are due to the non-equilibrium character of the depositing technique only, but also to the formation of salts during the immersion in SBF.

**Figure 8:**
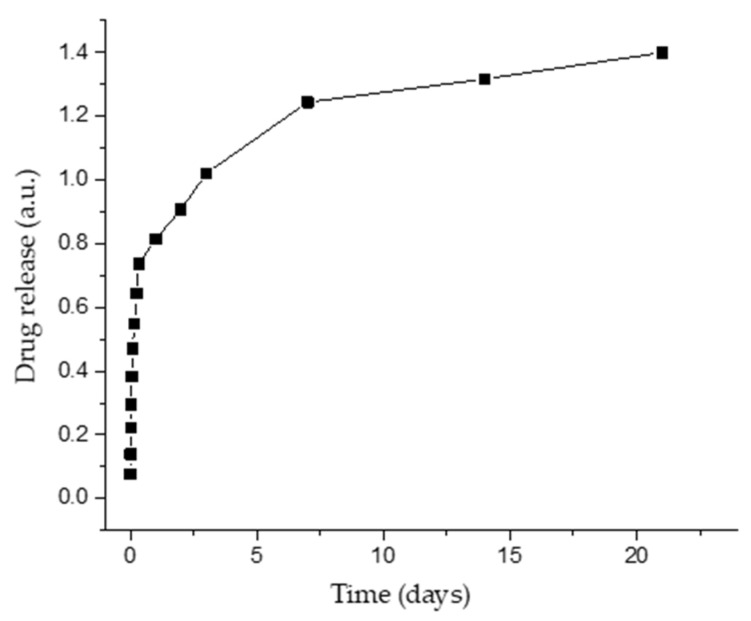
Drug release as function of time from BG+CIPRO/PMMA coatings.

SBF containing products released from samples was analyzed by UV-Vis. The drug release kinetics was studied for a prolonged period (21 days) and is displayed in Figure 8. The maximum wavelength (λmax), obtained by scanning all samples from 200 to 400 nm, was found to be 267 nm. As it can be observed, the antibiotic is released at a constant rate throughout the 21 days immersion interval. This process suggests the gradual dissolution of BG in SBF, simultaneously with the release of the antibiotic. There is a demand to design drug release systems that allow longer release periods, e.g., the treatment time for most patients with bone infection caused by bacteria is usually at least 4–6 weeks [43]. This represents an important reason in favor of local treatment compared to oral ones, as a result of prolonged storage of the drug in the MAPLE-deposited coating.

**Figure 9:**
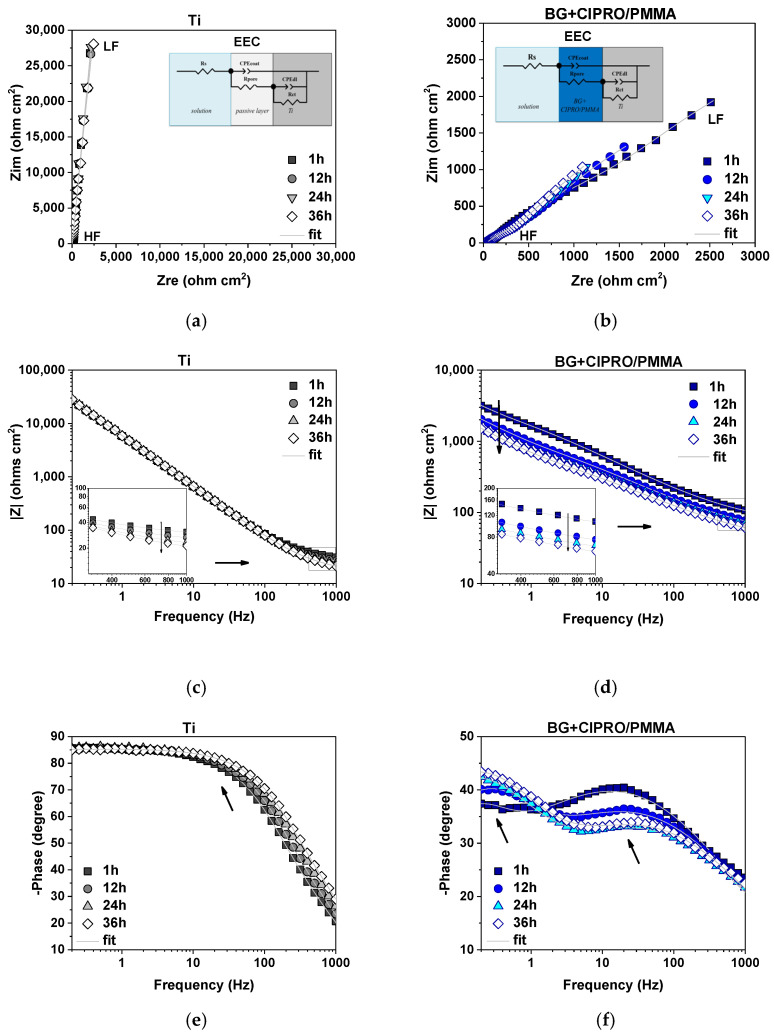
Nyquist and Bode amplitude and phase plots of Ti (**a**,**c**,**e**) and BG+CIPRO/PMMA-coated (**b**,**d**,**f**) Table 1*;* 12, 24, and 36 h of immersion in SBF at 37 °C (HF = high frequency range, HL = low frequency range, EEC = equivalent electrical circuit used for fitting the impedance data).

The electrochemical performance of the investigated systems during 36 h of immersion in SBF and their behavior after 1, 12, 24, and 36 h was monitored by EIS. Nyquist impedance plots of Ti and BG+CIPRO/PMMA-coated Ti (Figure 9a,b) showed a different AC polarization response with higher semicircles in the case of Ti, ascribed to higher charge transfer resistance. Moreover, a slight variation as a function of immersion time was observed when BG+CIPRO/PMMA structure was analyzed. Note the increase in semicircle diameter corresponding to the spectrum recorded after 36 h of immersion as compared with the initial EIS data, proving the higher corrosion resistance at the end of the immersion time.

As observed in Bode amplitude and phase plots, the impedance modulus varies significantly over the applied frequency range, more evident for BG+CIPRO/PMMA, revealing the dielectric properties of the investigated systems, observed at high-frequency range (HF). Data in the literature reported that the time constant present in the phase plot at medium frequencies (MF) provide information related to coating–solution interface, whereas the second time constant present in the low-frequency range (LF) is indicative of a corrosion process occurring at the substrate–solution interface [44]. The BG+CIPRO/PMMA-coated Ti is characterized by two well-defined time constants (indicated by arrows in Figure 9f). In the case of Ti, literature data reported the formation of a bilayer structure of oxide film, with thickness and characteristics as a function of the testing solution [45] and mainly formed of TiO_2_, Ti_3_O_5_, Ti_2_O_3_, and TiO oxides [46,47]. A two-time constant representative equivalent circuit was selected taking into consideration the results of XPS analysis showing the presence of the mentioned oxides and suboxides [46,48]. In the current study, the Bode phase plot characteristic to Ti sample shows a convolution for the two phases over the selected immersion time (indicated by the arrow in Figure 9e). Therefore, the recorded impedance data were fitted with the electrical equivalent circuit (EEC) presented in the inset of Figure 9a,b). In order to investigate the electrolyte–coating interface and also electrolyte–substrate interface, the following electrical components were considered: R_s_ (solution resistance), CPE_coat_ (coating capacitance), R_pore_ (resistance associated to the current flow through the pores generated by the coating defects), CPE_dl_ (double layer capacitance), and R_ct_ (charge transfer resistance). A constant phase element (CPE) was used to take into consideration the deviations from the ideal behavior [49]. As pointed out in reference [50], these deviations can be due to surface disorder or inhomogeneity, geometric irregularities, working electrode roughness, or porosity.

The electrochemical parameters obtained after the fitting procedure of impedance data with the mentioned EEC are given in Table 3. The associated relative error of each parameter and χ^2^ used as an indication of the goodness of fit is also presented. The values of relative error were lower than ~7% (calculated for R_ct_ in the case of BG+CIPRO/PMMA), whereas in the case of Ti the mentioned parameter could not be determined in the selected frequency range. Moreover, the low values obtained for χ^2^ parameter (7 × 10^−5^ ÷ 2 × 10^−4^) represent a validation of the fitting procedure.

The open-circuit potential (EOC) was recorded before each EIS measurement as a function of immersion time. A higher variation range was obtained for the Ti sample, showing the instability of the oxide layer formed when in contact with body fluids. As showed in reference [51], the mentioned layer is destroyed by Cl^−^ ions from SBF. The layer’s breakdown leads to Q_coat_ increase up to 36 h, when the higher value was recorded, as compared to the initial capacitance (Q_coat___Ti_ (1 h) = 19.89 μF s^(α−1)^ cm^−2^ and to Q_coat___Ti_ (36 h) = 19.05 μF s^(α−1)^ cm^−2^). Even though the spectra displayed by the Bode amplitude plot indicated a stable behavior in the middle-frequency range specific to the formed protective layer, the fitted parameters showed low values of R_pore_ with decreasing tendency over time, generating pathways for the electrolyte ingress. However, no corrosion process was detected. Moreover, a 5% increase (calculated at 0.2 Hz for 36 h immersion spectra as compared with the initial spectra) of EIS recorded data in LF range of Bode amplitude plot was observed. All the above results indicate the formation of new outer oxides that continuously rearrange after breakdown, having slightly higher protective properties against corrosion attack at the end of immersion.

On the other hand, BG+CIPRO/PMMA showed a more stable EOC (approximately −60 ÷ −80 mV) with less variation and a higher Rpore value as compared with Ti sample at the end of the test (R_pore___BG+CIPRO/PMMA_ (36 h) ~964 Ω cm^2^, whereas R_pore___Ti_ (36 h) ~58 Ω cm^2^). For both tested systems, solution resistance gradually decreased as the immersion time increased (as indicated in the inset of Figure 9c,d), with higher values obtained in the case of coated sample. A degradation over 36 h immersion of BG+CIPRO/PMMA coating was occurring, pointed out by the increase in Q_coat_ and associated αcoat parameter, which started at 0.55 and recorded a 0.51 value at the end of the test. A similar decreasing tendency was shown by the double-layer capacitance and αdl, respectively. Moreover, a 52% decrease of impedance modulus calculated at 0.2 Hz for 36 h immersion spectra as compared with the initial spectra was obtained. However, the charge transfer resistance recorded large values reaching 12,054 Ω cm^2^ after 36 h, indicating an improvement with time of protecting properties, thus delaying the contact between the substrate and corrosive ions. The decrease of maximum phase angle in MF range and the increase of phase angle in LF range suggest the BG dissolution in time, revealing the capacitive behavior of PMMA layer, which is in accordance with previous studies [17,51].

**Figure 10:**
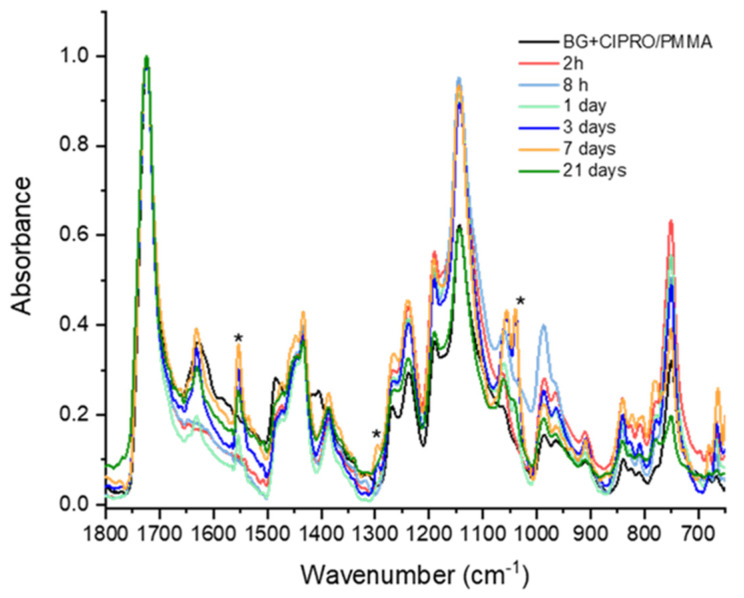
FTIR spectra of samples after different immersion times. * hydroxyl carbonate apatite peaks.

Figure 10 shows the IR spectra of the BG+CIPRO/PMMA thin films after 2 and 8 h, and 1, 3, 7, and 21 days of immersion in SBF, respectively. Transformations on the surface of the thin film were observed after the first day of immersion in SBF, indicating BG dissolution and the formation of a hydroxyl carbonate apatite layer on the film surface. The hydroxyl carbonate apatite peaks were found at 1553, 1300, and 1038 cm^−1^, respectively. The band at 1553 cm^−1^ was assigned to carbonate group absorption [52]. The peak around 1300 cm^−1^ was attributed to phosphate absorptions. The band at 1038 cm^−1^ corresponds to the asymmetric stretching of the P–O bond in (PO_4_)^3^^−^. This behavior is consistent with the apatite formation mechanism described in reference [53].

### 3.3. Biological Assays

**Figure 11:**
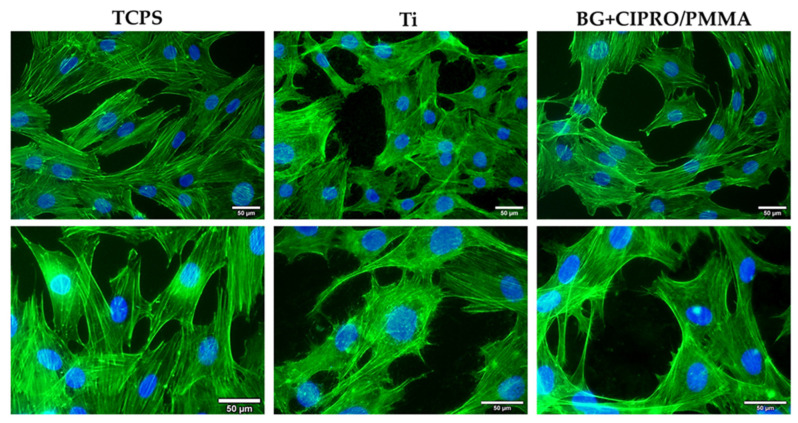
Fluorescence microscopy images of MC3T3-E1 osteoblasts (green: F-actin labelled with phalloidin-FITC; blue: nuclei labelled with DAPI; scale bar: 50 µm) after 24 h of incubation on TCPS control, bare Ti, and BG+CIPRO/PMMA samples.

As it can be observed in Figure 11, the fluorescence staining of actin filaments in MC3TE-E1 osteoblasts revealed almost similar cell densities for both uncoated and coated Ti samples compared to TCPS control after 24 h of incubation. The images revealed cell-to-cell junctions on tested samples, established by numerous lamellipodia and filopodia. These structures confirmed the good cell adhesion and migration on the coated surface, the osteoblasts having a structured actin cytoskeleton.

**Figure 12:**
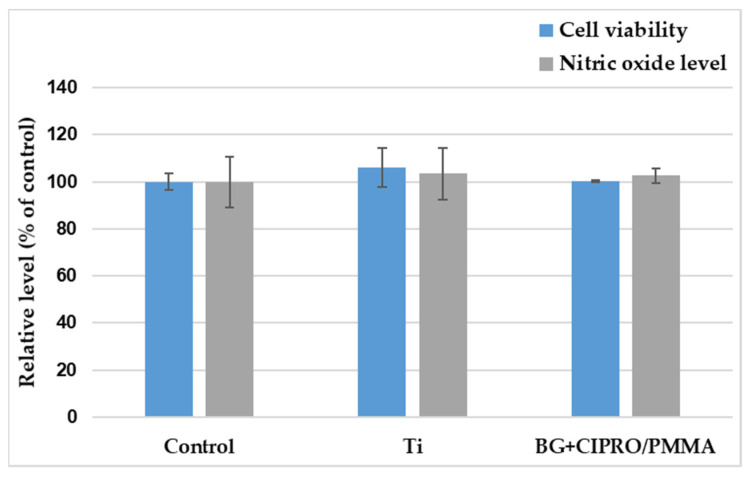
MTT (cell viability) and Griess (NO level). Results are presented as mean ± standard deviation of three independent experiments and represented as percentages of control.

The MTT assay results (Figure 12) were in good agreement with the fluorescence microscopy, the level of viable cells being almost similar for both control and samples. Their biocompatibility was also validated by Griess assay, as the level of nitric oxide did not increase, as compared to TCPS.

Gram-positive bacteria, such as *S. aureus* and *E. faecalis*, are dangerous and challenging infectious agents due to their continuously increasing resistance to antibiotics [54,55]. Among these, *S. aureus* is the most frequently isolated bacterial population from implant-associated infections [56]. Enterococci, including *E. faecalis*, are the second most frequently isolated Gram-positive bacteria [57].

Bacteria growth and adhesion on surfaces take place in two stages: (a) initial and reversible physical phase, and (b) irreversible chemical and cellular phase. In the first stage, many surface-related factors such as surface roughness and surface wettability affect the process. This stage usually takes 6 h and if bacteria attach to the surface during this stage, they can grow rapidly to form a biofilm [58].

**Figure 13:**
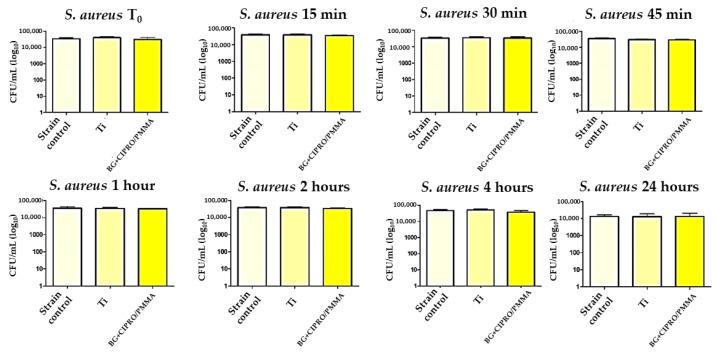
Evaluation of *S. aureus* viability in the presence of bare Ti and BG+CIPRO/PMMA samples.

**Figure 14:**
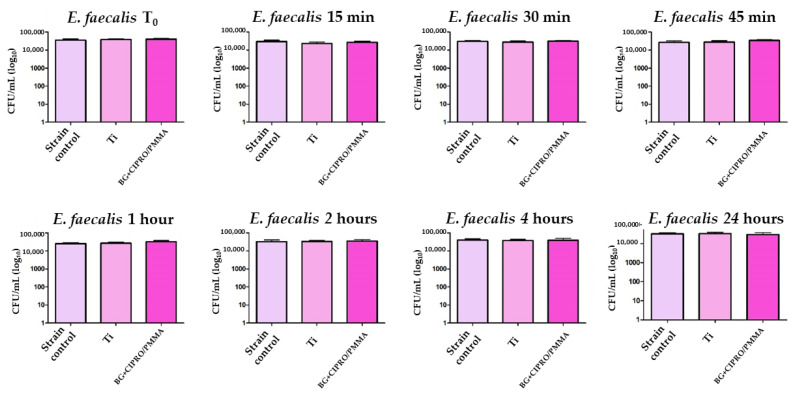
Evaluation of *E. faecalis* viability in the presence of bare Ti and BG+CIPRO/PMMA samples.

After 24 h of direct contact with bacterial strains in liquid media, Ti and BG+CIPRO/PMMA samples did not inhibit the growth of *S. aureus* (Figure 13) and *E. faecalis* (Figure 14).

*E. coli* represents one of the most common Gram-negative species implicated in the etiology of device-associated infections of endogenous origin [59], while *P. aeruginosa* is responsible for a great variety of medical device- and tissue biofilm-associated infections [60].

**Figure 15:**
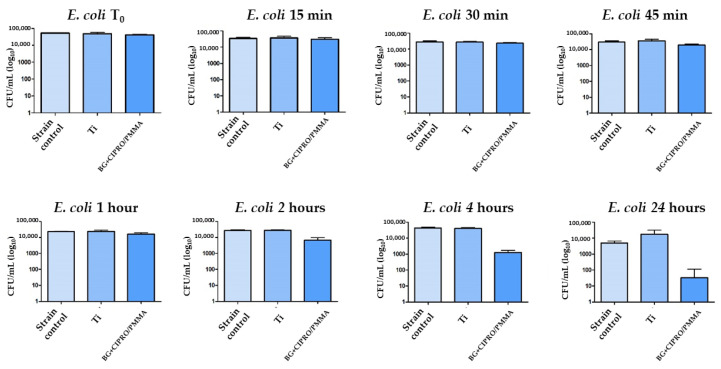
Evaluation of *E. coli* viability in the presence of bare Ti and BG+CIPRO/PMMA samples.

**Figure 16:**
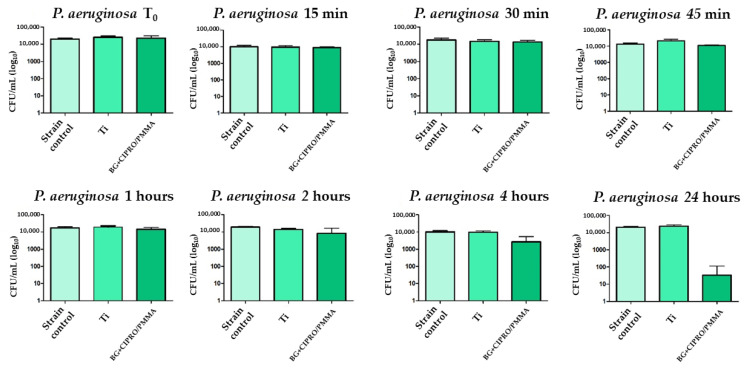
Evaluation of *P. aeruginosa* viability in the presence of bare Ti and BG+CIPRO/PMMA samples.

The bare Ti had no inhibitory effect on Gram-negative or Gram-positive bacteria, while the MAPLE-obtained coating interfered with the Gram-negative bacterial strain growth, a decrease of viable cell counts being noticed after the first 45 min of incubation. A 2-log reduction of the total viable cells was observed for BG+CIPRO/PMMA samples as compared to the control or Ti (Figure 15 and Figure 16).

Our results demonstrate that the antibiotic is released from MAPLE layers and is effective against Gram-negative bacteria starting with 45 min after the initial contact, while for Gram-positive strains we did not obtain an inhibition. Although ciprofloxacin is a second-generation quinolone with an extended spectrum including Gram-negative bacteria and some Gram-positive strains, the lack of an inhibitory effect against *S. aureus* and *E. faecalis* could be explained by the different structure of the cellular wall (e.g., the thick peptidoglycan of the Gram-positive strains delaying the intracellular accumulation of active concentrations). In the Gram-negative strains, the influx of fluoroquinolones is favored by porins expressed in their outer membrane, such as OmpF and OmpC in the case of *E. coli* [61,62].

## 4. Conclusions

The potential of BG+CIPRO/PMMA to bond to bone tissue is demonstrated by the presence of the carbonated hydroxyapatite layer.

The MAPLE-processed coating proved to be a good candidate for the design of efficient implant surfaces.

BG+CIPRO/PMMA coatings successfully inhibited Gram-negative bacterial growth with no harmful effects on MC3T3-E1 osteoblasts.

It can be concluded that our coating is promising in terms of its bioactivity and may find potential applications for bone repair and tissue engineering. In bone surgery, it offers more possibilities to adapt it with the bone metabolism activities when a rapid bone biomaterial interface is necessary.

## Figures and Tables

**Table 1:** Roughness values of thin films before immersion in SBF.

Sample	RMS (µm)	Ra (µm)
BG+CIPRO/PMMA_1	0.283	0.222
BG+CIPRO/PMMA_2	0.211	0.160

**Table 2:** Roughness values of thin films after 21 days of immersion in SBF.

Sample	RMS (µm)	Ra (µm)
BG+CIPRO/PMMA	0.330	0.277

**Table 3:** The open circuit potential (EOC) and EIS fitted parameters with the associated relative errors of Ti and BG+CIPRO/PMMA-coated Ti after 1, 12, 24, and 36 h of immersion in SBF at 37 °C.

Sample	Ti				BG+CIPRO/PMMA			
	1 h	12 h	24 h	36 h	1 h	12 h	24 h	36 h
E_OC_ (mV)	64.72	−29.75	8.28	−3.07	−63.79	−82.81	−62.57	−60.42
Rs (Ω cm^2^)	27.03 (0.28%)	22.51 (0.28%)	19.01 (0.34%)	16.51 (0.37%)	66.45 (0.68%)	44.99 (0.39%)	39.04 (0.36%)	34.19 (0.37%)
Qcoat (μF s^(α−1)^ cm^−2^)	19.89 (0.15%)	19.95 (0.14%)	20.25 (0.15%)	19.05 (0.16 %)	161.86 (0.26%)	257.06 (0.15 %)	318.27 (0.15%)	343.91 (0.14%)
α_coat_	0.96 (0.03%)	0.96 (0.03%)	0.96 (0.03%)	0.97 (0.03%)	0.55 (0.09%)	0.53 (0.05%)	0.52 (0.05%)	0.51 (0.05%)
*R*_pore_ (Ω cm^2^)	87.23 (2.01%)	74.98 (1.98%)	70.11 (2.32%)	58.34 (2.22%)	4337 (1.04%)	1735 (0.61%)	988 (0.52%)	964 (0.55%)
*Q*_dl_ (μF s^(α−1)^ cm^−2^)	9.90 (0.36%)	9.68 (0.35%)	9.13 (0.41%)	10.45 (0.37%)	191.39 (2.99%)	189.38 (0.97%)	284.68 (0.65%)	257.68 (0.66%)
α_dl_	0.93 (0.09%)	0.93 (0.09%)	0.93 (0.10%)	0.92 (0.09%)	0.89 (1.81%)	0.79 (0.58%)	0.73 (0.41 %)	0.73 (0.41%)
*R*_ct_ (Ω cm^2^)	-	-	-	-	7631 (7.43%)	7717 (2.74%)	11,401 (3.95%)	12,054 (3.97%)
*χ^2^*	2 × 10^−4^	2 × 10^−4^	2 × 10^−4^	2 × 10^−4^	3 × 10^−4^	8 × 10^−5^	7 × 10^−5^	7 × 10^−5^
